# MiR-17-5p and MKL-1 modulate stem cell characteristics of gastric cancer cells

**DOI:** 10.7150/ijbs.57338

**Published:** 2021-06-04

**Authors:** Zhou-Tong Dai, Yuan Xiang, Yuan-yuan Duan, Jun Wang, Jia Peng Li, Hui-Min Zhang, Chao Cheng, Qiong Wang, Tong-Cun Zhang, Xing-Hua Liao

**Affiliations:** 1 Institute of Biology and Medicine, College of Life and Health Sciences, Wuhan University of Science and Technology, Hubei, 430081, P.R. China.; 2 Department of Medical Laboratory, Central Hospital of Wuhan, Tongji Medical College, Huazhong University of Science and Technology, Hubei, 430014, P.R. China.; 3 Department of Gastrointestinal Surgery, Union Hospital, Tongji Medical College, Huazhong University of Science and Technology, Wuhan, Hubei, China.; 4 Key Laboratory of Industrial Fermentation Microbiology, Ministry of Education and Tianjin, College of Biotechnology, Tianjin University of Science and Technology, Tinajin, 300457, P.R. China.

**Keywords:** miR-17-5p, MKL-1, gastric cancer, stem cells, bioinformatics.

## Abstract

Effectively targeting cancer stem cells to treat cancer has great therapeutic prospects. However, the effect of microRNA miR-17/MKL-1 on gastric cancer stem cells has not been studied yet. This study preliminarily explored the mechanism of miR-17/MKL-1 in gastric cancer stem cells. Many previous reports have indicated that microRNA and EMT regulated cancer stem cell characteristics, and miR-17 and MKL-1 were involved as a critical gene in migration and invasion in the EMT pathway. Through RT-PCR, Western Blot, flow cytometry, immunofluorescence, sphere formation xenograft tumor assays and drug resistance, the role of miR-17-5p and MKL-1 on promoting stem cell-like properties of gastric cancer were verified *in vivo and vitro*. Next, MKL-1 targets CD44, EpCAM, and miR -17-5p promoter verified by luciferase assay and *ChIP*. Besides, the TCGA database analysis found that both miR-17-5p and MKL-1 increased in gastric cancer, and the prognostic survival of the MKL-1 high expression group was reduced. It is found that MKL-1 promotes expression by targeting miR-17, CD44 and EpCAM promoters. Besides, the TCGA database analysis found that both miR-17-5p and MKL-1 increased in gastric cancer, and the prognostic survival of the MKL-1 high expression group was reduced. These findings reveal new regulatory signaling pathways for gastric cancer stem cells, thus it give new insights on potential early diagnosis and/or molecular therapy for gastric cancer.

## Introduction

Gastric cancer was one of the malignancies seriously threatening the health of human beings, whose mortality ranked second among the malignant tumor diseases 1, 2. Although various methods of examination, surgical approaches, and chemotherapy protocols have continued to be improved, the five-year survival rate of gastric cancer patients remains 20% to 30% 3. Recent studies have shown that it was related to the presence of cancer stem cells (CSCs) in tumors. Cancer stem cells maintain the vitality of tumor cell populations through self-renewal and unlimited proliferation 4. The movement and migration ability of tumor stem cells makes tumor cell metastasis possible 5, 6. Cancer stem cells can stay in a dormant state for a long time and possess a variety of drug-resistant molecules, but are not sensitive to external physical and chemical factors that kill tumor cells 5, 6. Different sorts of specific cell surface markers were used to isolate individual CSCs from many solid tumors, such as breast cancer 7, colon cancer 8, liver cancer 9, pancreatic cancer 10, prostate cancer 11, stomach cancer 12, and ovarian cancer 13. There were many markers for gastric cancer stem cells (GCSCs). For example, CD44 12-14, CD133 15, and EpCAM 16 were often used as markers of traditional tumor stem cells. Those proteins related to the degree of differentiation of tumor cells, such as OCT4, Nanog, and Sox2, were usually used as markers of embryonic stem cells. They were also applicable to be employed to identify gastric CSCs 16. Thus, it could be seen that the research of cancer stem cells has become a new hot spot in oncology research. They had the self-renewal ability and differentiation potential and high tumorigenicity, which played an essential role in the growth, invasion, and metastasis of tumor cells. Besides, these cells were also in a dormant state for a long time, and then developed resistance to treatments such as radiotherapy and chemotherapy. These findings revealed that the basic research of gastric CSCs provided a new theoretical basis for finding specific targets for diagnosis and gene therapy of gastric cancer.

MicroRNA (miRNA) was a type of endogenous, non-coding small RNA in the body that was approximately 18-24 nucleotides in length. It interacted with the 3' untranslated region (3'UTR) of the target mRNA to cause degradation or translational inhibition of the target mRNA, regulated protein synthesis, and participated in a series of essential processes such as cell division, differentiation, and cell epithelial-mesenchymal transition 6, 17, 18. An increasing body of evidence suggested that miRNAs modulated the main features of tumor stem cells 18-20. In the tumor cells, miRNAs were involved in the regulation of stem cell expansion. For example, in the prostate cancer cell lines, overexpression of miR-34 inhibited tumor-initiating cell populations and significantly restrained the capability of sphere-forming of the tumor cells *in vitro* 21. While in the *in vivo* model, it also blocked tumor formation 21. Furthermore, miRNAs also played a part in the regulation of tumor differentiation. For instance, the expression of let-7 was not detected in the breast CSCs isolated from specimens of patients who had undergone chemotherapy, but when the cells were put in a differentiation-induced environment, the expression of let-7 had increased significantly 22. MiRNA was also involved in the regulation of cell epithelial-mesenchymal transition. Taken as examples, miR-30 family members participated in the regulation of epithelial-mesenchymal transition for tumor cells 23.

For GCSC, a large number of studies have shown that miRNA can regulate the characteristics of gastric cancer stem cells 24. Zeng et al. found that overexpression of miR-145 can inhibit the expression of CD44^+^ gastric cancer cells 25. At the same time, inhibiting miR-193a-3p will reduce cell viability and increase the number of apoptotic cells. miR-193a-3p may participate in the cisplatin resistance process of GCSC by regulating the mitochondrial apoptosis pathway 26. In addition, the inhibition of miR-196a-5p can also reduce the EMT pathway of GCSC through the SMAD4 signaling pathway, thereby reducing its invasion ability 27. In recent studies, it was found that miRNA-19b/20a/92a can promote the stem cell characteristics of gastric cancer 28. MiR-17-5p is also a member of this family. In renal cell carcinoma, inhibiting the expression of miR-17 can inhibit its formation spheroid ability, thereby reducing the stem cell characteristics of renal cell carcinoma 29. In colon cancer, the overexpression of miR-17 can promote the stem cell characteristics of cancer cells 30. However, there is no clear report in gastric cancer.

The gene for Megakaryoblastic leukemia translocation 1 (MKL-1), located on human chromosome 22, consisting of 807 amino acids, was a member of the family of cardiac-associated transcription factors 31. It was widely expressed in many cancers and regulated the migration and metastasis of tumor cells 32, 33. For instance, in the uterine leiomyosarcoma, a family of cardiac-associated transcription factors inhibited the growth of uterine leiomyosarcoma cells through activating the growth inhibitor-P21 34. Besides, the activation of hepatic stellate cells was regulated by the transforming growth factor signaling pathway 33. The family of myocardial-related transcription factors plays an essential role in the development of stem cells 35, 36. However, its effects on CSCs have not been found yet.

In this study, we found that MKL-1 not only promoted the stem cell characteristics of cancer cell MGC-803, but also played a regulatory mechanism through the miR-17/CD44/EpCAM pathway. In addition, it was also found that the overall survival of gastric cancer patients in the high-expressing MKL-1 group was poor. These findings reveal new regulatory signaling pathways for gastric cancer stem cells, which improved our current understanding of CSCs as well as informed potential early diagnosis and/or molecular therapy for gastric cancer.

## Materials and Methods

### Plasmids

The coding region sequences of MKL-1 genes were amplified by PCR using the cDNA of gastric cancer cells as the template (MKL-1:forward: 5'-ATGGCCCAATGGAATCAGCTACAGC-3', reverse:5'-TCACATGGGGGAGGTAGCGCACTCCGA-3') and inserted into pcDNA3.1 (Invitrogen, USA). The promoter sequences of CD44 and EpCAM were amplified by PCR (CD44 forward: 5'-aagcagccgtaatagggtttggatctaggtgttctag-3', CD44 reverse: 5'-CCAAACCCTATTACGGCTGCTTCTTAGTTGTGTGA-3'; EpCAM forward: 5'- agtggctgaggactagatccaTACAAGGATTTTTCAGTCCCA-3', EpCAM reverse: 5'-TGTTTGGGACTGAAAAATCCTTGTATGGATCTAGTCTCAGCCAC-3') and inserted into the pGL3-Promoter (Addgene, USA). The MKL-1 3'-UTR sequence was amplified by PCR (MKL-1 forward: 5'-ctctctggctcaagacggg-3', MKL-1 reverse: 5'-GTGTGTTTTTGTGTTT-3') and inserted into pmirGLO (Invitrogen, USA). The stable overexpression MKL-1 sequence was amplified by PCR (MKL-1 forward: 5'-GATTAGCTTGGTACGAATTCATGCCGCCTTTGAAAAGTCCAGC-3', MKL-1 reverse: 5'-AGAACTAGTCTCTCGAGGAATTCCTACAAGCAGGAATCCCAGTGCAGC-3') and inserted into pLVX (Invitrogen, USA). The stable knockdown MKL-1 sequence was amplified by PCR (MKL-1-shRNA1: 5'-CCGGCCTCACCAATGGAACCACTATCTCGAGATAGTGGTTCCATTGGTGAGGTTTTTG-3', MKL-1-shRNA2: 5'-CCGGCGCCACCTCTATCCTGCACAACTCGAGTTGTGCAGGATAGAGGTGGCGTTTTTG-3', MKL-1-shRNA3: 5'-CCGGTTGTGGGCCAGGTGAACTATCCTCGAGGATAGTTCACCTGGCCCACAATTTTTG-3') and inserted into pLKO.1 (Invitrogen, USA). The stable overexpression miR-17-5p sequence was amplified by PCR (miR-17-5p forward: 5'-CCGGGTCAGAATAATGTCAAAGTGCTTACAGTGCAGGTAGTGATATGTGCATCTACTGCAGTGAAGGCACTTGTAGCATTATGGTGACTTTTTG-3', miR-17-5p reverse: 5'-AATTCAAAAAGTCACCATAATGCTACAAGTGCCTTCACTGCAGTAGATGCACATATCACTACCTGCACTGTAAGCACTTTGACATTATTCTGAC-3') and inserted into pLVX (Invitrogen, USA). All plasmids were extracted from the plasmid extraction kit (Invitrogen, USA). The predicted recognition elements were mutated using a site-directed mutagenesis kit (Stratagene, USA).

### Cell culture

Gastric cancer cell lines SGC-7901, MGC-803, AGS, and 293T were obtained from the Chinese Academy of Sciences Cell Bank (Shanghai, China). The gastric cancer cell lines SGC-7901 and MGC-803 in the RPMI-1640 (Gbico, USA), culture medium containing 10% fetal bovine serum (Gbico, USA) and penicillin/streptomycin (Gbico, USA) and the cell line AGS in the DMED-F12 (Gbico, USA) culture medium containing 10% fetal calf serum (Gbico, USA) and penicillin/streptomycin (Gbico, USA) were cultured in an incubator (Senta, China) at 37°C with 5% CO_2_ and 95% air. The media were changed every two days.

### Transfection

According to the manufacturer instructions, Negative control mimic (NC mimic), miR-17-5p mimic (mimic), Negative control inhibitor (NC inhibitor), miR-17-5p inhibitor (inhibitor), Negative control siRNA (si-NC), siRNA-MKL1 (si-MKL1) were purchased from RIBOBIO biological. All plasmids were extracted with Endo Free Plasmid Midi Kit (CWBIO, China), all fragments and plasmids were transfected with lipofectamine 3000 (Invitrogen, USA) according to the manufacturer instructions.

### Lentivirus

For lentiviral plasmid pLKO.1 and pLVX, 5×10^6^ cells/ml cells of HEK-293T in the logarithmic growth phase were inoculated into a 10-centimeter culture dish. After the cells were attached, the stable overexpression or knockdown plasmid, which had been constructed previously, the plasmid GAG (Invitrogen, USA), and the packaging plasmid VSVG (Invitrogen, USA) were added for the co-transfection of the HEK-293T cells using Lipofectamine 3000 (Invitrogen, USA). When they were transfected for 24 hours, fresh DMEM cultures were exchanged. After transfection for 72 hours, the lentivirus solution was collected with a 0.45 μm filter into a cryovial. The Lenti-Pac HIV RT-PCR Titration Kit (GeneCopoeia, USA) was used to determine the virus concentration. The virus was added dropwise to gastric cancer cells MGC-803 and cultured in a 37°C and 5% CO_2_ saturated humidity incubator (Senta, China). After 72 hours of transfection, the cells were continuously screened for three days through puromycin. The screened cells were subjected to analyze and examine.

### Luciferase analysis

For the measurement of dual-luciferase, HEK-293T cells were seeded into the 96 (corning) orifice plate and cultured at 37 °C for 24 hours. The wild and mutant types of recombinant plasmids were transfected respectively into MGC-803 cells with Lipofectamine 3000 (Invitrogen, USA), which were placed into the incubator (Senta, China) for 48-hour further culture. Then the cells were collected and lysed. The luciferase activities of sea pansy and firefly of each hole of cells were detected using a dual-luciferase reporter assay kit (Promega, USA). This experiment was repeated three times.

### Tumor spheroid experiment of gastric cancer cells

The cells were digested with the trypsin (Gbico, USA) containing no EDTA and no phenol red. The cells were washed twice with PBS and once with serum-free medium DMEM/F12. 1000 cells were placed in the low-adhesion six-well plates (Corning, USA), cultured in the serum-free medium DMEM/F12, which involved 20 ng/mL EGF and 10 ng/mL bFGF. For the preliminary screening of experimental cell lines, a typical image after 21 days of serum-free culture was selected. For *in vivo* experiments, a typical image after 14 days of serum-free culture was selected. All images were observed and photographed by an inverted microscope (Olympus, Japan). The numbers of cells in each tumor spheroid cell were counted.

### Flow cytometry analysis

The expression of cell surface markers was detected by flow cytometry. The cells were digested by trypsin (Gbico, USA) without phenol red or EDTA. The cells were centrifuged and washed twice with PBS containing 5% BSA (Meilunbio, China). They were resuspended with PBS containing 5% BSA (Meilunbio, China). The cells were incubated on ice for 30 minutes with the corresponding antibody. Then cells were analyzed by the flow cytometer (BD, USA). The results were analyzed by FlowJo10.0 software. The following antibodies were used: CD44-FITC (BD, USA), EpCAM-PE (BD, USA).

### Enzyme-linked immunosorbent assay (ELISA)

ELISA was employed to detect the levels of Cytokeratin 14 (CK-14) and Cytokeratin 20 (CK-20) within the cells in each group using ELISA kit (R&D Systems, USA). The antigens were diluted using a carbonate-coated loading buffer. A total of 50-μL enzyme-labeled reagent was added to all wells and incubated at 37 °C for 1 h. Next, each reaction well was added with 100 μL tetramethylbenzidine substrate solution under conditions void of light. The reaction was then terminated through the addition of 50 μL stop buffer. Optical density was measured at 450 nm within a 15-min period using a microplate reader (Thermo, USA).

### Western Blot Analysis

The cells were lysed on ice for 30 minutes in RIPA lysis buffer (Meilunbio, China) containing phenylmethanesulfonyl fluoride (0.1 mg/mL). The supernatant was centrifuged to obtain the sample of the protein. The protein was quantified by a BCA kit (Meilunbio, China) with 10 μL of the supernatant taken. 40μg protein sample was loaded to 8% -12% gel electrophoresis SDS-PAGE. The protein was transferred to the polyvinylidene fluoride (PVDF) Membrane (Millipore, USA, ThermoFisher, USA). The PVDF membrane was blocked in a blocking solution by the TBST buffer (Meilunbio, China), which contains 5% skim milk powder (BD, USA) for one hour. The PVDF membrane was incubated with the primary antibody at 4 °C overnight and then incubated with the corresponding secondary antibody for 1 hour at room temperature. After being washed three times with TBST Buffer (Meilunbio, China), The PVDF membrane was detected by Western fluorescence assay Beyo ECL Plus (Beyotime, China, Meilunbio, China). The following antibodies were used: Anti-CD44 (1:2000, CST, USA), Anti-EpCAM (1:1000, CST, USA), Anti-MKL-1 (1:1000, CST, USA), Anti-GAPDH (1:1000, Abcam, USA).

### RT-PCR

The expression of mRNA and target miRNA in the cancer cells was detected by RT-PCR. The total RNA of the cells was extracted using the TRIzol (Invitrogen, USA). The samples with the ratio of OD260/OD280 of the RNA in the range of 1.8-2.1 were used for the subsequent reaction. The mRNA was reverse transcribed into cDNA by the reverse transcription kit (Takara, Japan). GAPDH was selected as the loading control for mRNA expression analyses. As to miRNAs, miRNAs were extracted using the miR Neasy Mini Kit. MiRNA reverse-transcription was conducted using the miR Script Reverse Transcription kit (Qiagen, Germany). U6 was selected as the loading control for miRNA expression analyses. The SYBR-green (Thermo, USA) detection system was used, and the reaction system was configured according to the instructions for PCR reaction and fluorescence signal collection. 94 °C for 4 min, 94 °C for 30 s, 58 °C for 30 s, and 72 °C for 30 s with 39 cycles. Data processing analyses Folds=2-^ΔΔ^Ct. Three separate replicates were performed for each experiment. The miRNA and U6 primers were designed by Ribobio (Ribobio, China). The following mRNA primers were used:

GAPDH: forward: 5′-GGAGCGAGATCCCTCCAAAAT-3′, reverse: 5′-GGCTGTTGTCATACTTCTCATGG-3′.

CD44: forward: 5′-GCCGCTTTGCAGGTGTATTC-3′, reverse: 5′-GCTTTCTCCATCTGGGCCAT-3′.

EpCAM: forward: 5′-CCATGTGCTGGTGTGTGAAC-3′, reverse: 5′-GAAGTGCAGTCCGCAAACTT-3′.

MKL-1: forward: 5′-ATGCCGCCTTTGAAAAGTCCA-3′, reverse: 5′-TCTTCCGTTTGAGATAGTCCTCT-3′.

Ki67: forward: 5′-AGAAGAAGTGGTGCTTCGGAA-3′, reverse: 5′-AGTTTGCGTGGCCTGTACTAA-3′.

### RNA Pull-Down Assay

RNA pulldown assay was carried out using Magnetic RNA-Protein Pull-Down Kit (Thermo, USA). The label of the Target RNA was bound to Streptavidin Magnetic Beads. The RNA-bound beads were added to the cell nuclear lysate. Then, the eluted proteins were detected by Western Blot analysis.

### RNA Immunoprecipitation (RIP)

RNA-protein-antibody complexes were captured using Protein A/G (Thermo, USA). RNA was eluted by adding TRIzol directly to magnetic beads and isolated as per the manufacturer instructions. cDNA was synthesized using HiScript® II 1st Strand cDNA Synthesis Kit (Vazyme, China) and analyzed by RT-PCR.

### CCK⁃8 assay

CCK8 assay (Dojindo, Japan) was used to detect cell proliferation and resistance. Each group of cells was seeded into 96-well plates. The number of cells in each well was about 2×104. After cultured for 0 days, 1day, 2days, and 3 days, 10μL of reagent CCK-8 were added, and the cells were placed in an incubator for 2 hours. The enzyme-linked immunosorbent assay was applied to measure the absorbance at 450 nm using a Varioskan TM LUX microplate reader (Thermo, USA) to draw a growth curve. For the drug resistance experiment, Different concentrations of chemotherapeutic drugs were added, and then the cells were further cultured for 24 hours. The enzyme-linked immunosorbent assay was applied to measure the absorbance at 450 nm using a Varioskan TM LUX microplate reader (Thermo, USA). The drugs used included cisplatin (Sigma, USA) and paclitaxel (Sigma, USA), respectively. Six replicate wells were set for each group.

### Human tumor xenograft model

Animal experiments were conducted following the guidelines of the Laboratory Animal Center of Wuhan University of Science and Technology. This experiment used 18 male BALB/c nude mice, which were 4 weeks old and about 15 grams of weight. They were purchased from Beijing Huafukang Experimental Animal Co., Ltd., and they were raised in the Experimental Animal Center of Wuhan University of Science and Technology. Cells with stable overexpression of miR-17-5p, MKL-1, and its control cells in the logarithmic growth phase were digested with trypsin (Gbico, USA) without phenol red and EDTA. Then resuspend it in PBS containing 50% Matrigel (BD, USA). The cell suspension 2x10^6^(cells/ml) was injected subcutaneously into the dorsal side of the nude mice BALB/C. The mice were sacrificed 4 weeks later. Biochemical and histopathological analyses were performed on blood and tumor samples collected from mice. The Laboratory Animal Center has approved the research plan of Wuhan University of Science and Technology.

### Histology analysis

The tissues were fixed, dehydrated, and paraffin-embedded in the 10% formalin to make tissue sections of about 5 microns through H&E staining. Samples were observed using a microscope (Olympus, Japan).

### Immunohistochemistry

The tissues were fixed, dehydrated, and paraffin-embedded in the 10% formalin to make tissue sections of about 5 microns. Xylene was used to dewax them twice for 15 minutes each time. The sections were followed by incubation in 3% H2O2 (Sigma-Aldrich, St. Louis, MO, USA) at 37 °C for 30 min and boiling in 0.01 M citric acid buffer at 95 °C for 20 min and then blocked with serum working solution at 37 °C for 10 min and incubated with diluted primary antibodies at 37 °C for 2 h. Then, the sections were incubated with Horseradish Peroxidase (HRP) conjugated secondary immunoglobulin G (IgG) antibody and counterstained with hematoxylin (Meilunbio, China) at room temperature for 4 min. The sections were observed using a microscope (Olympus, Japan). The following antibodies were used: Anti-CD44 (1:2000, CST, USA), Anti-EpCAM (1:1000, CST, USA), Anti-MKL-1 (1:1000, CST, USA), Anti-GAPDH (1:1000, Abcam, USA).

### Immunofluorescence and confocal imaging

Immunofluorescence was used to detect the expression of CD44, EpCAM, and MKL-1 in the cells. The cells were grown on a coverslip of 10 centimeters and were fixed in a 4% paraformaldehyde solution followed by permeabilization with 0.5% Triton X-100 (Sigma, USA). Block with goat serum in a dark box for 1 day. The cells were incubated with the corresponding primary antibody overnight at 4°C. Cy3-conjugated anti-rabbit IgG (Abcam, USA) and FITC-conjugated anti-mouse IgG (Abcam, USA) were used as secondary antibodies. After incubation, these cells were incubated for two hours with the nuclei stained by DAPI (Thermo, USA). Ultimately, the samples were coverslipped with a fluorescence microscope.

### Chromatin immunoprecipitation (ChIP) assays

Follow the instructions of the Simple ChIP® Enzymatic Chromatin IP Kit (CST, USA). Formaldehyde was added to the cells to reach a final concentration of 1% of chromatin cross-linking. Moreover, collect cells for nuclear processing and chromatin shearing. The samples were divided into three groups during the chromatin immunoprecipitation process. Target protein antibody, IgG antibody, and no antibody were added to each group at 4 °C overnight. ChIP-grade protein G agarose beads were added to each group and incubated; the beads were eluted and cross-linked, and the DNA was purified by a spin column for PCR.

### Bioinformatics

TCGA database was a gene chip-based database and integrated data extraction platform. In this database, it could set the conditions for extracting data according to needs (https://cancergenome.nih.gov/). R software with the limma package was used to analyze TCGA clinical data. UALCAN online analysis tool was used to analyze the prognosis of miR-17 and MKL-1 from TCGA (http://ualcan.path.uab.edu/).

### Statistics

Univariate and multivariate analyses were performed on the experimental data using SPSS 17.0 and GraphPad 7.0 software. Statistical analysis was determined by two-tailed student's t-tests unless otherwise stated. Asterisks indicate statistical significance (*P< 0.05; **P< 0.01; ***P< 0.001). The survival curve of the mice was calculated using the method of Kaplan-Meier. Furthermore, the Wilcoxon test was used for comparison. Unless the mean ± SEM was specified in the legend, data were reported as mean ± SD.

## Results

### Expression of tumor stem cell characteristics in different cells

**Figure 1:**
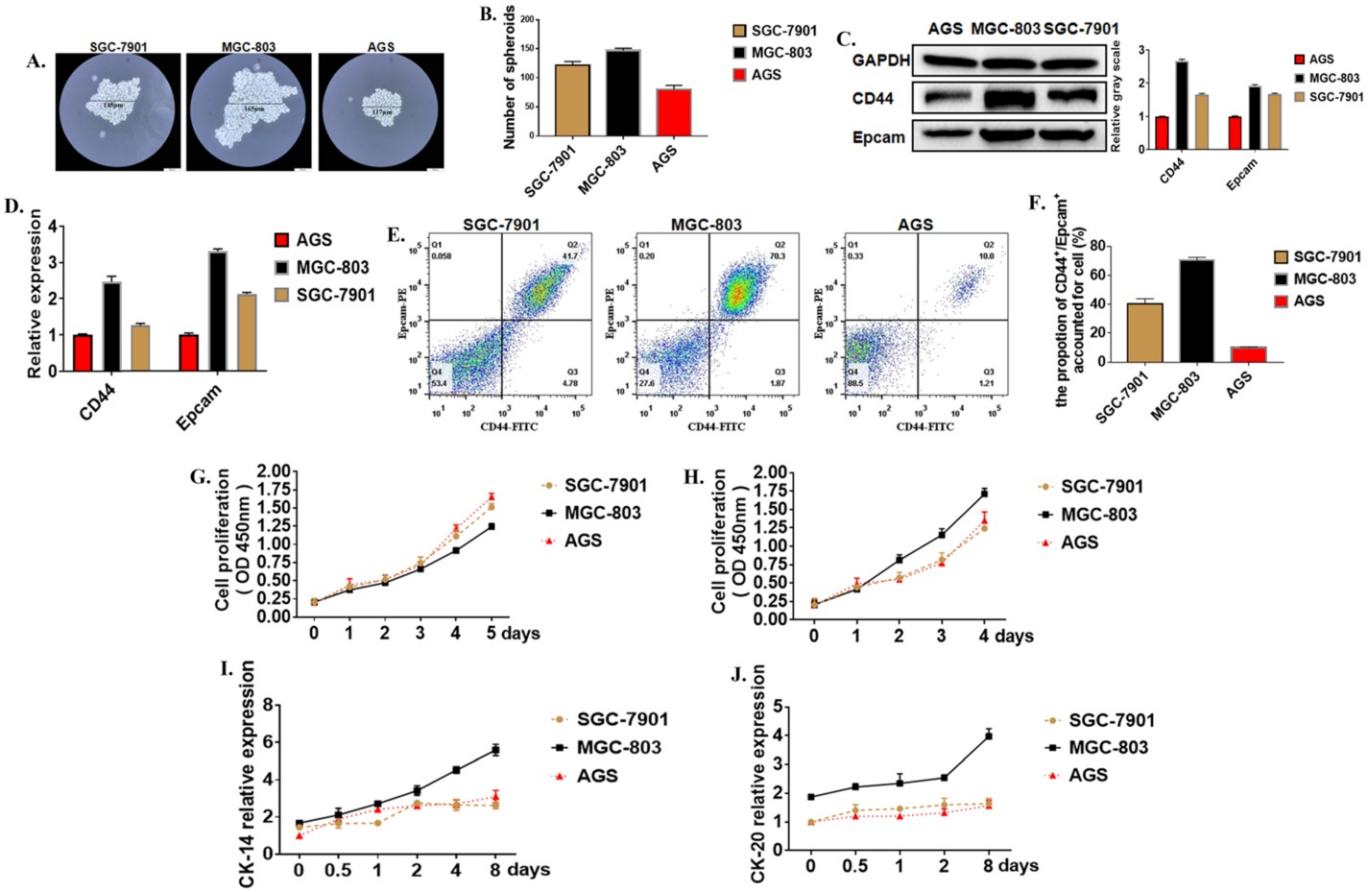
** Tumor stem cell characteristics in different cells. A.** Typical images of tumor cells in three different gastric cancer cell lines (SGC-7901, MGC-803, AGS cell lines were cultured in serum-free medium for 21 days). **B.** The number of tumor spheroids after 21 days of culture in serum-free medium. **C.** Expression of GCSC markers CD44 and EpCAM proteins in MGC-803 cell line using Western Blot analysis (GAPDH was used as an internal control). **D.** RT-PCR for the expression of GCSC markers CD44 and EpCAM in the three cancer cell lines (GAPDH was used as an internal control). **E.** Expression of CD44 and EpCAM in three cancer cell lines by flow cytometry. **F.** The bar chart of propotion of CD44^+^/Epcam^+^ accounted for cell. **G.** Cell proliferation curves of three cells tested under serum-free culture conditions using the CCK-8 kit.** H.** Tumor cell proliferation curves for the cultivation by adding 10% fetal bovine serum-containing medium after 48 hours of culture in serum-free medium. **I, J.** Expression of markers CK-14 and CK-20 in three cancer cell lines using ELISA. Data represents the mean ± SEM (n=3-6/group, significantly different as compared to each control, *P<0.05, **P<0.01), unless otherwise noted.

Cancer stem cells (CSCs) are a special kind of stem cells found in many tumor tissues in recent years. They have the ability of self-renewal and differentiation. They can differentiate into tumor cells to form the primary tumor and metastasis. Moreover, cancer stem cells are not sensitive to chemotherapy and radiotherapy. Formation Spheroid ability is an important method for the identification of tumor stem cells *in vitro*. Three cell lines with different levels of differentiation were used to test the strength of cancer stem cells. The results showed that after 21 days of culture in serum-free and low-adsorption culture dishes, the number of tumor globular cells enriched in the MGC-803 cell line was higher than the SGC-7901 and AGS cell lines (**Figure 1A, B**). The molecular surface markers CD44 and EpCAM were determined to identify whether it was a tumor stem cell. Western Blot showed that the expression of CD44 in the MGC-803 cell line was significantly higher than the SGC-7901 and AGS cell lines (**Figure 1C**). It was demonstrated at the post-transcriptional level by RT-PCR results (**Figure 1D**). Besides, flow cytometry showed that (70.53%±1.07) of CD44^+^ and EpCAM^+^ positive cells were expressed in the MGC-803 cell line, while in the SGC-7901 line, there was about (40.63%±1.92) of the double-positive cells. However, only (10.03%±0.26) of double-positive cells in the AGS cell line (**Figure 1E, F**). CCK-8 proliferation experiments showed that the proliferation ability of the MGC-803 tumor spheroid cell line was not significantly different from the SGC-7901 and AGS cell lines (**Figure 1G**) when it was cultivated using RPMI 1640 medium. However, compared with before adding serums, the increase in OD450 of MGC-803 was higher than that of the SGC-7901 and AGS cell lines (MGC-803=0.403±0.013, SGC-7901=0.134±0.007, AGS=0.101±0.275). (**Figure 1H**). Moreover, tumor stem cells have a strong ability to differentiate. When isolated tumor spheroid cells were cultured in a medium with fetal bovine serum, the differentiation markers of the three cell line cells CK-14 and CK-18 increases with time (**Figure 1I, J**), and MGC-803 showed a higher differentiation potential. All these proved that MGC-803 had significant stem cell characteristics.

### MKL-1 promotes the stem cell characteristics of gastric cancer cells

**Figure 2:**
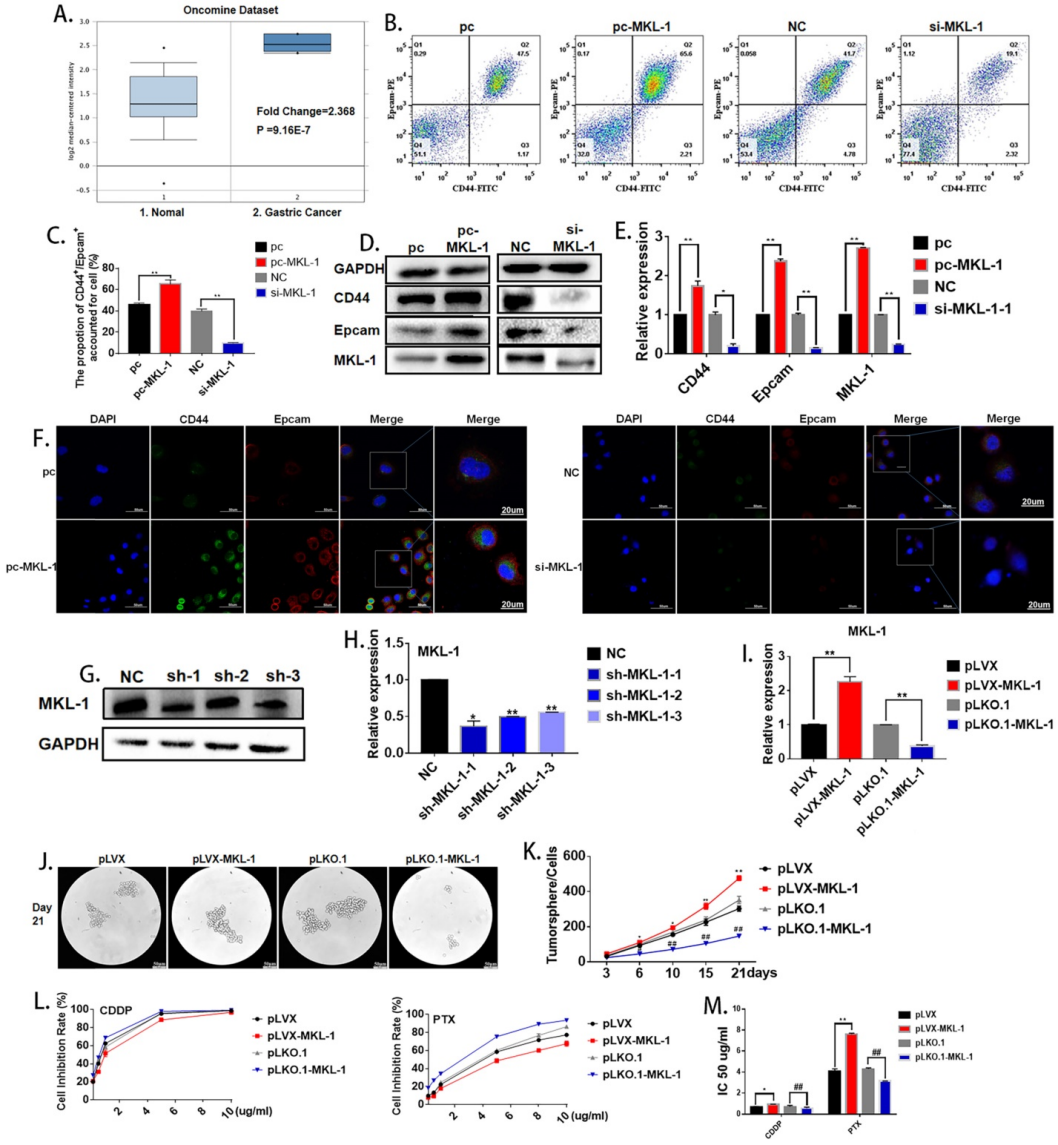
** Effect of MKL-1 on the stem cell characteristics of gastric cancer cells. A.** Expression of MKL-1 in normal tissues and gastric cancer cells from the Oncemine database. **B.** Expression of CD44 and EpCAM for the transient transfection overexpression (pc-MKL-1) and knockdown of the MKL-1 (si-MKL-1) in MGC-803 cell lines respectively using Flow cytometry analyses. **C.** The bar chart of propotion of CD44^+^/Epcam^+^ accounted for cell. **D, E.** Expression of CD44 and EpCAM for the transient transfection overexpression (pc-MKL-1) and knockdown (si-MKL-1) of the MKL-1 in MGC-803 cell lines respectively using Western Blot analyses and RT-PCR. **F.** Expression and location of CD44 and EpCAM using immunofluorescence analyses. **G, H.** Comparison of the expression of MKL-1 after stable knocking down MKL-1 from MGC-803 cell line at three different knockdown sites analyzed by Western Blot and RT-PCR. **I.** Stable overexpression (pLVX-MKL-1) and knockdown (pLKO.1-MKL-1) efficiencies of MKL-1 in MGC-803 by lentivirus infection using RT-PCR.** J.** Significant images of tumor spheres of the MGC-803 cell line with stable overexpression and knockdown of MKL-1 respectively after 21 days of serum-free culture. **K.** Number of tumor spheres in different cell lines under serum-free culture conditions. **L.** Inhibition rate of tumor sphere cells treated with different concentrations of cisplatin and paclitaxel for 24h after 21 days of serum-free cultivation. **M.** IC 50 of the cell line transfected with overexpression and knockdown MKL-1, after CDDP and PTX treatment. Data represents the mean ± SEM (n=4-6/group, significantly different as compared to each control, the control group of * is pLVX, the control group of # is pLKO.1. *P<0.05, **P<0.01, #*P*<0.05, ##*P*<0.01). Statistical comparisons among multiple groups were done by one-way ANOVA with Bonferroni correction, unless otherwise noted.

Oncomine database (https://www.oncomine.org) was used in order to explore the possible role of MKL-1 in gastric cancer cells. The search conditions were: Gene: MKL-1; Analysis Type: Cancer vs. Normal Analysis; Cancer Type: Gastric Cancer. It was found that MKL-1 was significantly up-regulated in gastric cancer cells (**Figure 2A**). According to reports 24, 25, the abnormal expression of MKL-1 in humans was closely related to tumor migration and differentiation, so it was speculated that MKL-1 affected the characteristics of GCSCs. In order to verify the effect of MKL-1 on the stem characteristics of GCSCs, the plasmids pcDNA3.1-MKL-1 and siRNA-MKL-1 for MKL-1 overexpression and knockdown were constructed. Flow cytometry analysis showed that overexpression of the MKL-1 could significantly increase the expression of GCSCs markers CD44 and EpCAM (**Figure 2B, C**). Western Blot results showed that the expression of the markers of GCSCs, CD44, and EpCAM, increased similarly to the results obtained by flow cytometer (**Figure 2D**). RT-PCR and immunofluorescence results gave the same tendency as the analyses described above (**Figures 2E, F**). In order to verify the long-term effects of MKL-1 on the formation of GCSCs, three knockdown MKL-1 cell lines with different mutation sites were constructed. The cell line with the highest knockdown efficiency was sh-MKL-1-1 (Figures **2G, H**). At the same time, stable overexpression of MKL-1 cell line MGC-803-pLVX-MKL-1 was also constructed. The overexpression and knockdown efficiencies of the two kinds of cell lines were shown in the figure (**Figure 2I**). Cell lines were cultured using a serum-free medium culture, and the results showed that in MGC-803-pLVX-MKL-1, the tumor formation speed and size were significantly higher than MGC-803-pLVX (**Figures 2J, K**). The tumor formation ability of MGC-803-pLKO.1-MKL-1 was significantly slower than that of the negative control group. In order to verify the effect of MKL-1 on different anticancer drugs, we have found that after 21 days of serum-free culture and 24 hours of treatment with CDDP, MGC-803-pLVX-MKL-1 with the overexpression of MKL-1 slightly increased the resistance of CDDP. For cell lines treated with paclitaxel for 24 hours, the knockdown of MKL-1 significantly reduced the drug resistance of the paclitaxel, and the drug resistance was positively correlated with time (**Figure 2L, M**). These results indicate that high expression of MKL-1 promoted the stem cell characteristics of gastric cancer.

### MiR-17-5p enhanced characteristics of the GCSCs

**Figure 3:**
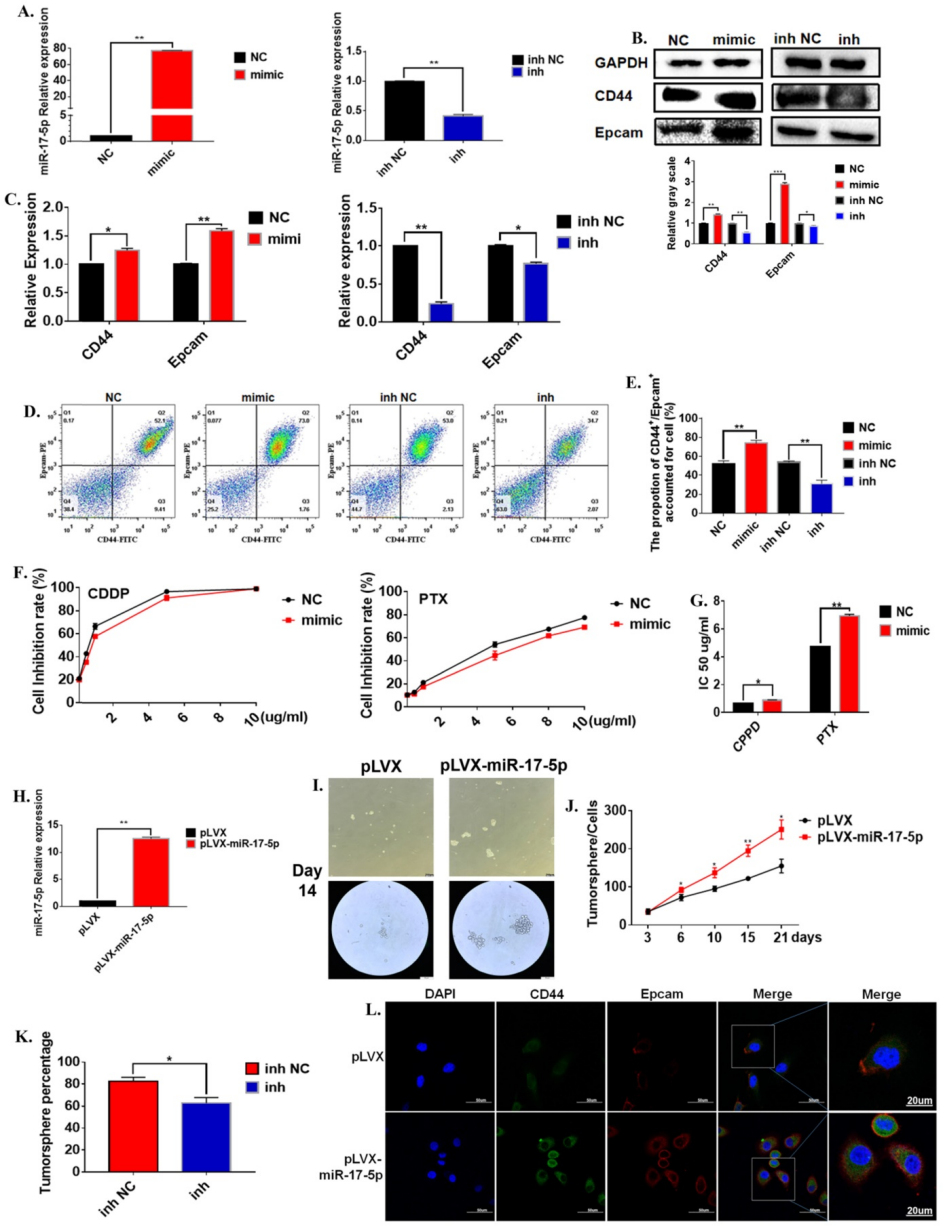
** The effect of miR-17-5p on the stem cell characteristics of GCSCs. A.** Relative expression of overexpression of miR-17-5p in MGC-803 cell line using RT-PCR (Relative expression of inhibitory efficiency of miR-17-5p in MGC-803 cell line using RT-PCR). **B.** Expression of CD44 and EpCAM using Western Blot analysis in MGC-803 cells transfected with miR-17-5p mimic and its negative control, miR-17-5p inhibitor and its negative control respectively.** C.** Expression of CD44 and EpCAM using RT-PCR.** D.** Expression of CD44 and EpCAM using flow cytometry. **E.** The bar chart of propotion of CD44^+^/Epcam^+^ accounted for cell. **F.** The inhibition rate of different concentrations of CDDP and PTX on transfected MGC-803 cells after the cells being treated for 24 hours (CCK-8 method). **G.** IC 50 of the cell line transfected with miR-17-5p mimic, after CDDP and PTX treatment. **H.** Relative overexpression efficiency of miR-17-5p in MGC-803 cell line was analyzed by RT-PCR. **I.** Typical images of gastric cancer tumor sphere cells with stable over-expression of miR-17-5p and the negative controls after 14 days of culture in serum-free medium. **J.** Curves of the number of Gastric cancer tumor cells with stable overexpression of miR-17-5p (Cultured in serum-free medium for 21 days). **K.** Percentage of tumor sphere cells after transfection with miR-17-5p inhibitor 3 days. **L.** Immunofluorescence analysis of CD44 and EpCAM expression of MGC-803-pLVX and MGC-803-pLVX-miR-17-5p. Data represents the mean SEM (n=4-6/group, significantly different as compared to each control, *P<0.05, **P<0.01). Statistical comparisons among multiple groups were done by one-way ANOVA with Bonferroni correction, unless otherwise noted.

In order to further verify the regulation of miR-17-5p on the characteristics of gastric cancer stem cells. We evaluated the effects of changes in miR-17-5p on the characteristics of cancer stem cells from multiple aspects such as cell viability, morphology, cancer stem cell markers. The mature miR-17-5p mimic was transferred to the cell line to test its efficiency. The results show that miR-17-5p mimic could significantly up-regulate the expression level of miR-17-5p, and miR-17-5p inhibitors significantly down-regulated the expression level of miR-17-5p in the MGC-803 cell line (**Figure 3A**). The results of the Western Blot experiment for the 72 hours cultivation showed that compared to the negative control group, the overexpression of miR-17-5p in the MGC-803 cell line significantly promoted the expression of the stem cell marker CD44 on the surface of the MGC-803 cells. The expression of EpCAM was also increased slightly. The transfection with the inhibitor of miR-17-5p could reduce the expression of CD44 and EpCAM in the MGC-803 cell line (**Figure 3B**). At the same time, the results of RT-PCR and immunofluorescence were consistent with the Western Blot (**Figure 3C**). Flow cytometry results showed that 72h after transfection with miR-17-5p analogs, the expression of CD44 and Epacm increased in the MGC-803 cell line (74.07±1.80) compared with the control group (52.47±1.77). The opposite situation occurred after transfection with the inhibitor (inh NC: 54.00±0.81, inh: 31.20±2.05). It was worth noting that the expressions of CD44 and EpCAM in the MGC-803 cell line were reduced after transfection with the mimic of lip3000 compared with the blank (**Figure 3D, E**). When two chemotherapeutic drugs, cisplatin, and paclitaxel, were added into the medium of MGC-803 cells 72 hours after transfection of miR-17-5p mimic, respectively. The number of surviving MGC-803 cells transfected with the miR-17-5p analog was significantly increased compared with the control group (**Figure 3F**). Meanwhile, the IC50 of the cell line transfected with miR-17-5p which the Cisplatin (CDDP) treatment group or Paclitaxel (PTX) treatment group to the drug was significantly higher than that of the control group (**Figure 3G**). In order to determine the long-term effects of miR-17-5p on the stem cell characteristics of cancer cells, two stable transfected cell lines of MGC-803-pLVX and MGC-803-pLVX-miR-17-5p were constructed and confirmed by RT-PCR (**Figure 3H**). The two stably transfected cell lines were also cultured in a serum-free medium for 21 days. MGC-803-pLVX-miR-17-5p began to form cell spheroid within 3 days. However, in the MGC-803-pLVX, spheroid began to form after 4-5 days of cultivation. When cultured for about 14 days, the spheroid cells were completely formed for the MGC-803 cell line with stable expression of miR-17-5p, and the center density increased. However, the spheroid structure of the control group was relatively loose, and the number of cells in the spheroid was significantly less (**Figure 3I**). After 21 days of cultivation, the number of tumor spheroid cells was counted. The number of spheroids of MGC-803-pLVX-miR-17-5p was significantly higher than that of the MGC-803-pLVX (**Figure 3J**). MGC-803-pLVX-miR-17-5p was cultured after three days in a serum-free medium, the miR-17-5p inhibitor and its negative control were transfected. The percentage of spheres in the inhibitor group was significantly reduced (**Figure 3K**). Immunofluorescence analysis showed that the cellular localisation of CD44 and EpCAM did not change, but the intensity of the fluorescent signal increased for 21 days of cultivation of MGC-803 cell line with stable expression of miR-17-5p, which indicated that overexpression of miR-17-5p increased the expression of CD44 and EpCAM of the cancer cells (**Figure 3L**). These findings indicate that miR-17-5p modulated the characteristics of GCSCs of the MGC-803 cell line *in vitro*.

### MKL-1 targets promoter regions of CD44, EpCAM, and miR-17

**Figure 4:**
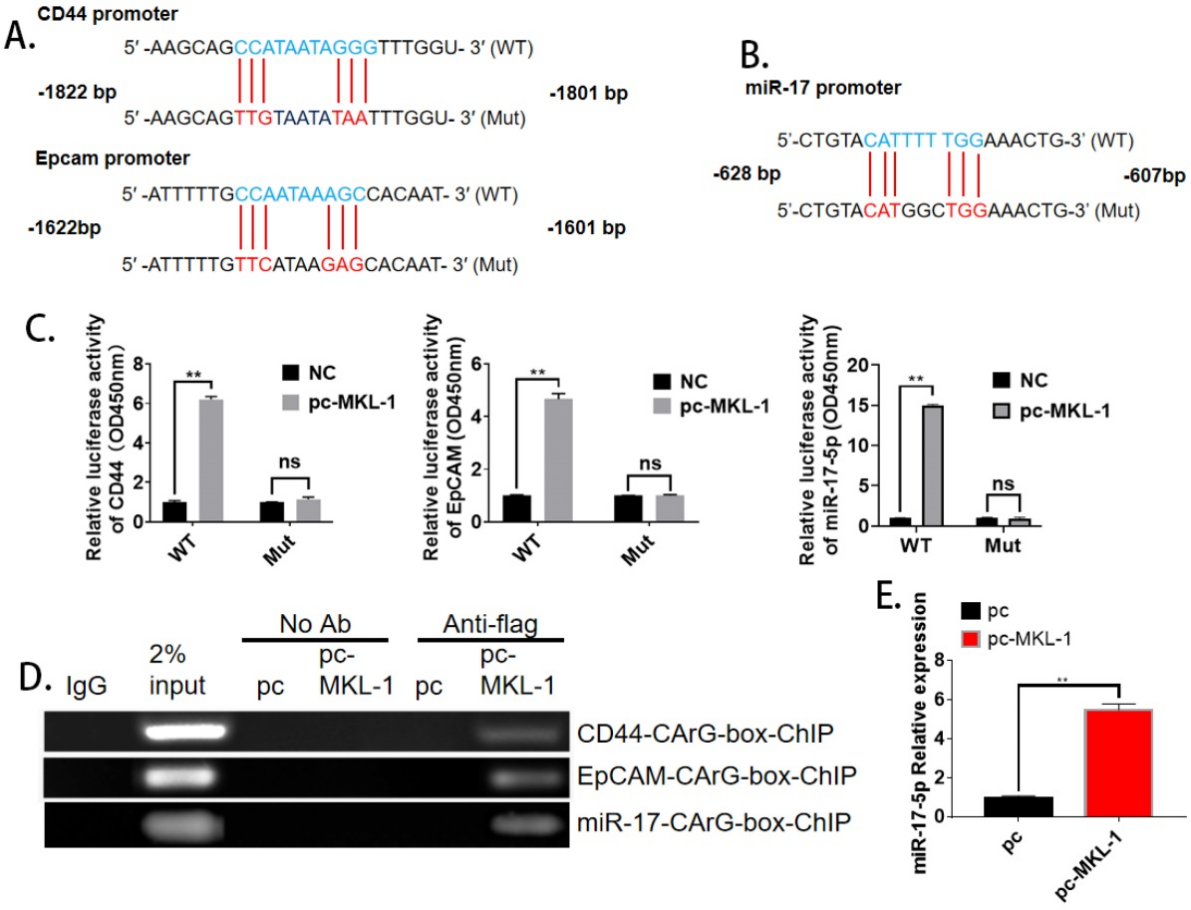
** MKL-1 targets promoter regions of CD44 and EpCAM.** The promoter mutation sites of CD44 and EpCAM, including the putative CArG box binding site of MKL-1. **B.** The promoter mutation sites of miR-17, including the putative CArG box binding site of MKL-1. **C.** Relative expression of co-transfected MKL-1 with CD44 Luc, EpCAM Luc and miR-17 Luc plasmids were analyzed by Dual luciferase assay. **D.** MKL-1 binds to the promoters of CD44, EpCAM and miR-17 using ChIP analysis. **E.** Relative expression of miR-17-5p after overexpression of MKL-1 by using RT-PCR. Data represents the mean ± SEM (n=4-6/group, significantly different as compared to each control, *P<0.05, **P<0.01). Statistical comparisons among multiple groups were done by one-way ANOVA with Bonferroni correction, unless otherwise noted.

With the analysis of bioinformatics software (TargetScan), it was found that both CD44, EpCAM, and miR-17 have MKL-1 specific DNA recognition sequence CArG box (CC ( A/T)_6_ GG) binding sites (**Figure 4A, B**). Therefore, MKL-1 could activate the transcription and expression of target genes of CD44, EpCAM, and miR-17. Subsequently, plasmids with CD44, EpCAM, and miR-17 of the CArG box sequence promoters were constructed. Luciferase analysis results showed that the luciferase activity of the overexpression of MKL-1 was significantly higher than that of the control group, The overexpression of MKL-1 did not affect the fluorescence activity of CD44, EpCAM, or miR-17 promoter mutant luciferase reporter plasmid (**Figure 4C**). At the same time, the ChIP primers of CD44 and EpCAM promoters were designed, and the ChIP results further demonstrated that MKL-1 bind to the CD44, EpCAM, and miR-17 promoters (**Figure 4D**). Meanwhile, overexpression of MKL-1 will significantly increase the expression of miR-17-5p (**Figure 4E**).

### MiR-17-5p targets the 3'UTR of MKL-1

**Figure 5:**
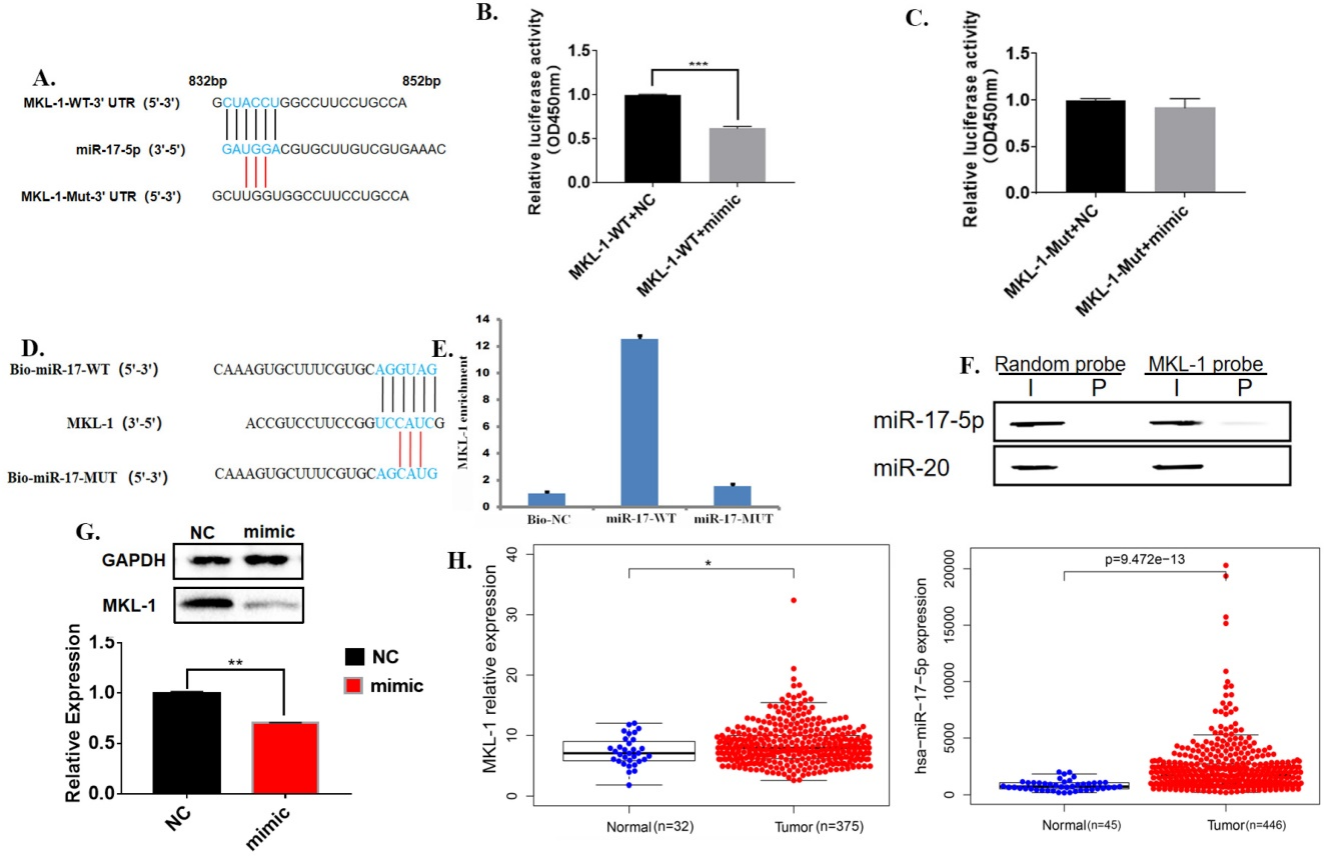
** MiR-17-5p targets the 3'UTR of MKL-1. A.** The promoter mutation sites of MKL-1, including the putative binding site of miR-17-5p. **B., C.** Relative expression of co-transfected miR-17-5p mimic with MKL-1-3'UTR-WT-Luc plasmid and MKL-1-**3'UTR**-MUT-Luc plasmid were analyzed by Dual luciferase assay. **D.** Schematic diagram of miR-17-5p-specific probe design. **E.** RIP experiment results verify that miR-17-5p binds to MKL-1. **F.** Western Blot experiment to verify that miR-17 and miR-20 specific probes using RNA pull down. **G.** The expression of MKL-1 after overexpression of miR-17-5p mimic by using Western Blot and RT-PCR. **H.** Heatmaps show the expression of MKL-1 and miR-17-5p in tumors and normal tissues in TCGA database. The data were statistically analyzed with t-test. Data represents the mean SEM (n=3-6/group, significantly different as compared to each control, *P<0.05, **P<0.01), unless otherwise noted.

Bioinformatics software analysis found that the 3'UTR of MKL-1 has 6 bases complementary to miR-17-5p, so it was speculated that they had a binding site (**Figure 5A**). Further, the dual luciferase reporter gene experiment showed that the luciferase activity of the transfected miR-17-5p mimic was significantly reduced compared with the control group (**Figure 5B**). Moreover, miR-17-5p mimic did not affect the fluorescent activity of the MKL-1 3'UTR mutant luciferase reporter plasmid (**Figure 5C**). Rip showed that high expression of miR-17-5p could improve the MKL-1 expression (**Figure 5D, 5E**). Meanwhile, other specific probes of the miR-17-5p family were designed and found that only miR-17-5p specific probes bind MKL-1 by using RNA pulldown (**Figure 5F**). After transfecting mature miR-17-5p into the cells, the results of WB and RT-PCR both showed that miR-17-5p could inhibit the expression of MKL-1 compared with the control (**Figure 5G**). Clinical information from the TCGA database was extracted. It was found that both miR-17-5p and MKL-1 were the high expression in gastric cancer patients (**Figure 5H**). It indicates that there was still a way to be discovered, which inhibits the regulation of miR-17-5p on MKL-1.

### The effects of miR-17-5p and MKL-1 on the characteristics of GCSCs *in vivo*

**Figure 6:**
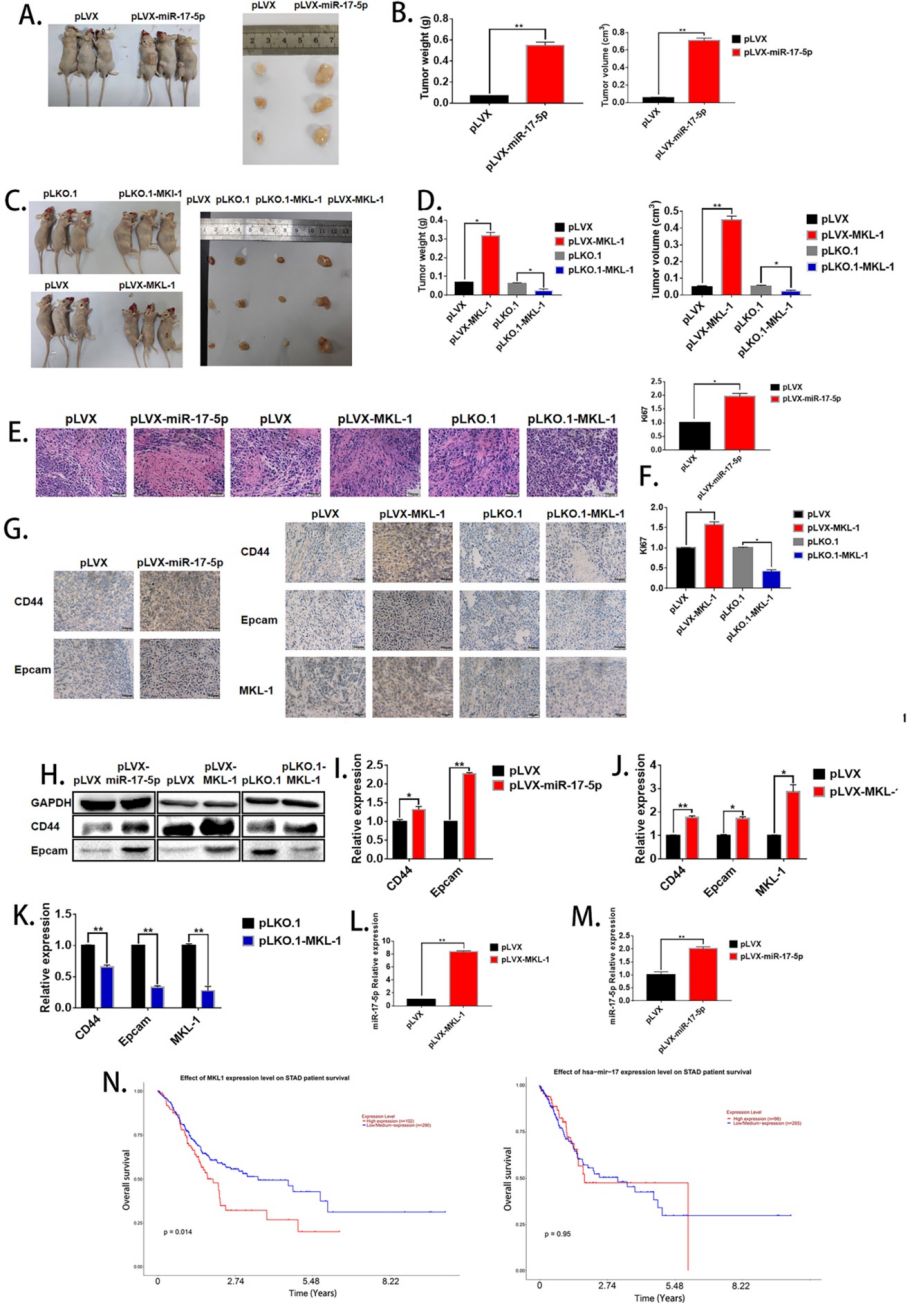
** The effect of miR-17-5p and MKL-1 on characteristics of tumor stem cell *in vivo*. A, C.** Typical picture of tumor obtained after nude mice were sacrificed after 28 days. **B, D.** Tumor volume and weight graph of tumor xenografts in mice. **E.** Image of nude mice subcutaneous tumors stained by H&E. **F.** Relative expression of Ki67 in xenograft tumorigenesis of nude mice by RT-PCR. **G.** IHC images for the detection of CD44, EpCAM and MKL-1 in subcutaneous tumors in nude mice.** H.** Expression of CD44, EpCAM and MKL-1 using Western Blot analyses in subcutaneous tumors. **I., J, K.** Expression of CD44, EpCAM and MKL-1 in subcutaneous tumors using RT-PCR analyses. **L.** Expression of miR-17-5p in subcutaneous tumors (MKL-1 overexpression group). **M.** Expression of miR-17-5p in subcutaneous tumors. **N.** The survival curves of patients with gastric cancer associated with miR-17-5p or MKL-1 in the TCGA database by using UALCAN. Data represents the mean SEM (n=4-6/group, significantly different as compared to each control, *P<0.05, **P<0.01). Statistical comparisons among multiple groups were done by one-way ANOVA with Bonferroni correction, unless otherwise noted.

In order to evaluate the effect of miR-17-5p and MKL-1 on the stem cell characteristics *in vivo*, the stable MGC-803 cell lines with overexpression of miR-17-5p (Figure 2H), overexpression, and knockdown of MKL-1 were constructed (Figure 3I), respectively. When the mice were sacrificed 28 days, the tumor tissues formed with the overexpression of MKL-1 and miR-17-5p were larger in mass and volume than that of the control group (**Figures 6A, B, C, D**). Besides, after H&E staining of tumor tissues, compared with the negative group, the cancer cells in the overexpressed MKL-1 and miR-17-5p group were more irregular in shape, larger in nucleus and tissue sizes, and darker in color with more obvious nuclear heterogeneity. (**Figure 6E**). Meanwhile, the expression of Ki67 in tumor tissues was determined. It was found that the overexpression group was significantly higher than the control group (**Figure 6F**). It was worth noting that the results of IHC showed that compared with the negative group, the expressions of CD44 and EpCAM in the tumor tissue groups with the overexpression of miR-17-5p and MKL-1 increased, while their expression of the knockdown MKL-1 group was lower than that of the negative group (**Figure 6G**). Total RNA and protein of the mouse tumor tissues were extracted, and the expression of CD44, EpCAM, and MKL-1 was detected by RT-PCR and Western Blot, respectively. The results showed that compared with the tumor tissues *in vivo*, the tumor tissues have more expression of CD44 and EpCAM (**Figures 6H, 6I, 6J, 6K**). At the same time, RNA was extracted from the tissue to detect miR-17-5p. The results showed that overexpression of MKL-1 could promote the expression of miR-17-5p (**Figure 6L**). In addition, the expression of miR-17-5p in the precursor of miR-17-5p was increased compared to the control group, but the fold change relative to the *in vitro* experiment was reduced (**Figure 6M**). This may be caused by circRNA or lncRNA competing with miRNA *in vivo*.

To determine the effect of miR-17-5p and MKL-1 expression on the prognosis of gastric cancer patients, the patient's expression of MKL-1, miR-17-5p, and survival time, survival status was used by UALCAN tools to draw the survival curve. TCGA clinical data was compiled, and it found that patients with high expression of MKL-1 showed poor prognosis (**Figure 6N**). However, the expression of miR-17-5p was not related to the survival time of patients in the TCGA database (**Figure 6N**).

## Discussion

Gastric cancer was a common malignant tumor of the digestive system. Because the early symptoms were not obvious, it was often aware in the middle and late stage, and the prognosis was poor 37. Although the total effective rate of clinical treatment current has been improved, the mortality rate of patients has not been effectively reduced 38. The exploration of pathways and mechanisms of gastric cancer prognosis was still the current research hotspot. Studies have confirmed that GCSCs were firmly related to the drug resistance, metastasis, and recurrence of gastric cancer and were the root cause of gastric cancer recurrence 39. GCSCs had the characteristics of self-renewal, differentiation and proliferation, and high drug resistance. They were present in gastric tumor tissues, and their strong DNA repair ability led to tumor formation, recurrence, and invasion 40. In this study, we analyzed the *in vitro* stem cell characteristics of different gastric cancer cell lines as a research model and found that stem cell characteristics vary widely among different cell lines. MGC-803 expressed the highest cancer stem cell characteristics by using morphology, stem cell markers, and differentiation markers.

MKL-1 was a newly discovered serum response factor (SRF) synergistic transcription factor that binds to a specific sequence of the target gene CArG cassette (CC(A/T)_6_GG box) 41. Through bioinformatics software, it was found that the tumor stem cell markers CD44 and EpCAM promoter had MKL-1 binding site CArG box. MKL-1 was widely expressed in tissues and regulated the transcription and expression of genes related to cell migration, invasion, and apoptosis involving in the occurrence and development of cancer 42, 43. It has previously been reported that MKL-1 plays an essential role in the differentiation of mammary epithelial cells and epithelial-mesenchymal transition (EMT) 44-46. Besides, it has been previously reported that the transformation of non-stem cells into CSC occurs through the acquisition of EMT and invasive mesenchyme 47. GCSCs induced by EMT had a strong tendency to enhance gastric cancer formation. EMT was also a critical link in the invasion and metastasis of gastric cancer. In summary, the EMT process enhanced the invasion and metastasis ability of gastric cancer cells, it promoted the formation of GCSCs. GCSCs participated in the initiation and maintenance of EMT. There was no absolute causal relationship between them, and they were commonly affected by multiple factors, such as the impact of drugs and the environmental. MKL-1 was confirmed to be involved in the EMT process of cells. In this study, the novel signaling pathway model was proved that regulated stem cell characteristics of tumor cells, and it was found that CSCs regulated the expression of gastric stem cell characteristics through the MKL-1. The high expression of MKL-1 significantly promoted the characteristics of cancer stem cells.

MiRNAs were an evolutionarily conserved small non-coding RNA consisting of 18-24 nucleotides that negatively regulated gene expression by binding to the target gene mRNA 3'UTR to mediate post-transcriptional silencing of the target gene. Several studies have confirmed that miRNAs were abnormally expressed in a variety of CSCs, some of which up-regulate the role of oncogenes, and some express down-regulation of the role of tumor suppressor genes. Studies have shown that miR-146b-5p attenuated the stem cell characteristics of glioma stem cells by targeting the HuR/lincRNA-p21/β-catenin signaling pathway 48. In liver CSCs, miR-122 was not expressed or expressed very little. It inhibited the invasion of liver CSCs through Smad/TGF-β signaling pathway and reduced recurrence 49. GCSCs were treated with nanotechnology-mediated miR-34a targeted transport 50. The miR-17-92 cluster contained six members, including five highly conserved seed regions, including miR-17, miR-18a, miR-19a/b, miR-20a, miR-92a 51. The miR-17-92 cluster was a multifunctional oncogenic miRNA cluster that played an important role in tumor angiogenesis and tissue development 52, 53. The miR-17-92 cluster was reported to be highly expressed in human endothelial cells, and some studies have found that the miR-17-92 cluster had anti-apoptotic effects 54. In GCSC, as mentioned in the introduction, there have been many studies that have proven that miRNA can regulate stem cells of gastric cancer 24-27. Moreover, miR-17-5p can promote other types of cancer stem cells 28-30. In our study, the regulation of miR-17-5p on MKL-1 and GCSCs was investigated. It was found that miR-17-5p and MKL-1 promoted the stem cell characteristics of GCSCs. On the other hand, it has been verified that MKL-1 can target the promoters of CD44, EpCAM, and miR-17-5p. In addition, mature miR-17-5p can inhibit the 3'UTR effect of MKL-1 *in vitro*. However, this inhibitory effect is eliminated *in vivo*. The reason for this may be due to the presence of circRNA or lncRNA as a sponge to dilute this effect. *In vitro* experiments, we used a mature miRNA mimic, which has a high overexpression fold. And this effect is diluted *in vivo*. It may also be related to it. Besides, Clinical information from the TCGA database was extracted. It was found that both miR-17-5p and MKL-1 were the high expression in gastric cancer patients. This also indirectly indicates that there is another way to inhibit the regulation of MKL-1 by miR-17-5p *in vivo*.

In previous studies, MKL-1 55/miR-17 30, 56 have been shown to be involved in the EMT pathway of cancer cells. And miR-17 has been proven to promote miR-17-5p can promote kidney cancer 29, colon cancer 30 stem cell characteristics. But their relationship is not known. The results showed that MGC-803 has the highest tumor stem cell characteristics among gastric cancer cell lines. And MKL-1 can target the miR-17-5p promoter to promote the characteristics of gastric cancer stem cells. Besides, it was also found that miR-17-5p can inhibit the expression of MKL-1 *in vitro*. Finally, by analyzing the extensive sample data in the TGCA database, the relationship between the expression level of miR-17-5p and the survival rate of gastric cancer patients was examined. It was found that patients with high expression of MKL-1 had a poorer prognosis. However, in the TCGA database, the expression of miR-17-5p has nothing to do with the survival time of the patient. This study provides further insights into the interpretation of the regulatory pathways of gastric cancer stem cells. In conclusion, we propose that the MKL-1-miR-17-CD44 pathway is an important determinant of the progression of gastric cancer stem cells, and targeting this pathway may provide new therapeutic opportunities for the treatment of gastric cancer.

## Abbreviations

MKL-1: Megakaryoblastic leukemia 1
SRF: serum response factor
FITC: fluorescein isothiocyanate
DAPI: 4',6-diamidino-2-phenylindole
RT-PCR: real-time PCR
WB: Western Blot
NC: negative control
CDDP: Cisplatin
PTX: Paclitaxel
TCGA: The Cancer Genome Atlas Program
GEO: Gene Expression Omnibus
