# MiR-17-5p and MKL-1 modulate stem cell characteristics of gastric cancer cells: Erratum

**DOI:** 10.7150/ijbs.73846

**Published:** 2022-04-26

**Authors:** Zhou-Tong Dai, Yuan Xiang, Yuan-yuan Duan, Jun Wang, Jia Peng Li, Hui-Min Zhang, Chao Cheng, Qiong Wang, Tong-Cun Zhang, Xing-Hua Liao

**Affiliations:** 1 Institute of Biology and Medicine, College of Life and Health Sciences, Wuhan University of Science and Technology, Hubei, 430081, P.R. China.; 2 Department of Medical Laboratory, Central Hospital of Wuhan, Tongji Medical College, Huazhong University of Science and Technology, Hubei, 430014, P.R. China.; 3 Department of Gastrointestinal Surgery, Union Hospital, Tongji Medical College, Huazhong University of Science and Technology, Wuhan, Hubei, China.; 4 Key Laboratory of Industrial Fermentation Microbiology, Ministry of Education and Tianjin, College of Biotechnology, Tianjin University of Science and Technology, Tinajin, 300457, P.R. China.

In our paper 1, there were 3 errors due to the mishandling of the order of the figures when we were revising the manuscript. We regret that we did not detect these errors before publication.

We all authors feel sorry for this carelessness. The corrected text and figures are shown below. These revisions do not alter the scientific conclusions of the manuscript.

(1) In the abstract, "Besides, the TCGA database analysis found that both miR-17-5p and MKL-1 increased in gastric cancer, and the prognostic survival of the MKL-1 high expression group was reduced." This sentence appears 2 times. Therefore, the second occurrence should be omitted in the abstract.

(2) The figure legend in Figure 5G. “The expression of MKL-1 after overexpression of miR-17-5p mimic by using Western Blot and RT-PCR.” should be changed to “The expression of MKL-1 after overexpression of miR-17-5p mimic by using Western Blot (Western Blot and relative gray scale)”. Also, on page 2286, paragraph 1, line 3 is revised to “......the results of Western Blot showed that......”.

(3) The images of Figures 2B and Figure 6G were incorrectly used. The correct Figures 2B and Figure 6G are as follows:

## Figures and Tables

**Figure 2:**
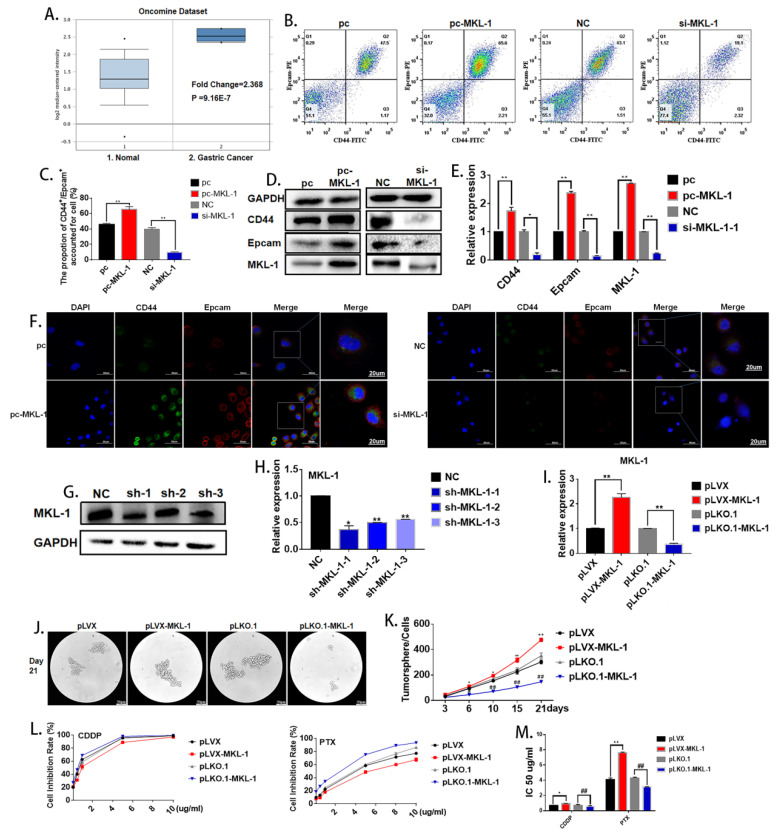
Corrected figure.

**Figure 6:**
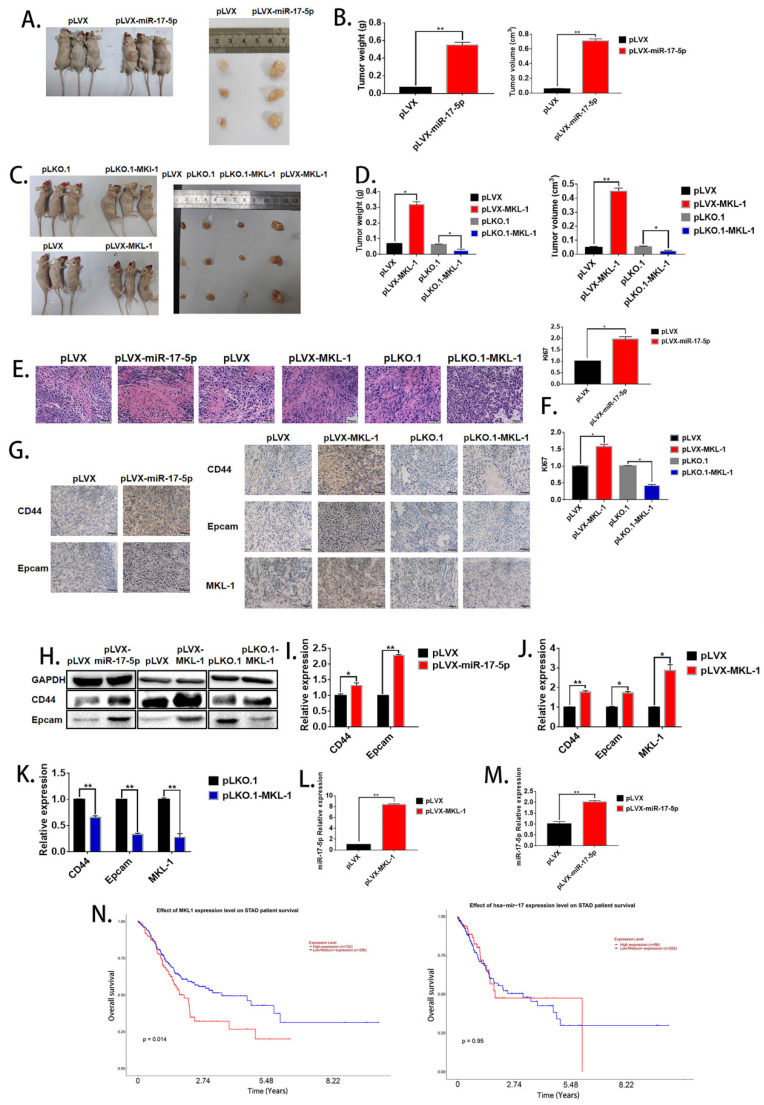
Corrected figure.
